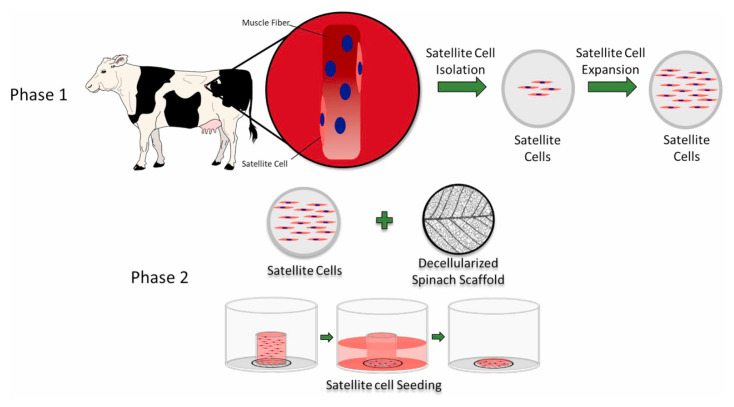# Current technologies, regulation, and future perspective of animal product analogs — A review

**DOI:** 10.5713/ab.23.0029

**Published:** 2023-05-02

**Authors:** Seung Yun Lee, Da Young Lee, Jae Won Jeong, Jae Hyeon Kim, Seung Hyeon Yun, Ermie Mariano, Juhyun Lee, Sungkwon Park, Cheorun Jo, Sun Jin Hur

**Affiliations:** 1 Division of Animal Science, Division of Applied Life Science (BK21 Four), Gyeongsang National University, Jinju 52828, Korea; 2 Institute of Agriculture and Life Science, Gyeongsang National University, Jinju 52828, Korea; 3 Department of Animal Science and Technology, Chung-Ang University, Anseong 17546, Korea; 4 Department of Food Science and Biotechnology, Sejong University, Seoul 05006, Korea; 5 Department of Agricultural Biotechnology, Center for Food and Bioconvergence, and Research Institute of Agriculture and Life Science, Seoul National University, Seoul 08826, Korea

**Keywords:** Cultured Meat, Insect, Livestock, Meat Analog, Mycoprotein, Plant-based

## Abstract

The purpose of this study was to investigate the recent development of meat analog, industrialization, and the related legal changes worldwide. Summarizing the current status of the industrialization of meat analog, studies on plant-based meat, mycoprotein, and edible insects were mainly conducted to investigate their sensory properties (texture, taste, flavor, and color resembling meat), nutritional and safety evaluations, acquisition method of meat alternatives, and commercialization. Cultured meat is mainly studied for developing muscle satellite cell acquisition and support techniques or materials for the formation of structures. However, these technologies have not reached the level for active industrialization. Even though there are differences in the food categories and labeling between countries, it is common to cause confusion or to relay false information to consumers; therefore, it is important to provide accurate information. In this study, there were some differences in the food classification and food definition (labeling) contents for each country and state depending on the product shape or form, raw materials, and ingredients. Therefore, this study can provide information about the current research available on meat alternatives, improve regulation, and clarify laws related to the meat analog industry, which can potentially grow alongside the livestock industry.

## INTRODUCTION

The definition of meat analog or meat alternative refers to the replacement of the main ingredient with an ingredient other than meat, which is also called a meat alternative, meat substitute, fake meat, mock meat, and imitation meat [1]. These products are principally made of pulses (mainly soy), cereals, or fungus protein, but the utilization of new protein sources, such as insects and seaweed, has been considered [2]. The history of meat analog is not only old, but also has been extensively studied. In 2013, Dutch scientist Mark Post introduced meat made from cultured meat for the first time [3], greatly increasing interest in the development of meat alternatives. The most representative meat analog materials are plant-based and insect-based meat analogs, mycoprotein, or cultured meat. Currently, the most notable meat analog material is cultured meat, and many researchers around the world are conducting studies to industrialize cultured meat. Although many researchers and companies have announced their development of cultured meat, there are few cases of industrialization. The reason is that although cultivating cells is not a very difficult technology, producing large quantities of cultured meat for human consumption is potentially challenging because of its high technical difficulty. On the other hand, meat analog manufactured using isolated soy protein, gluten, or insect protein is not only already sold on the market, but also has growing sales. This may be because plant-based or insect-based materials, unlike cultured meat, are easy to obtain, relatively easy to manufacture into products, and are already authorized as food ingredients. Although the type of material and the difficulty level of the production technology are different, meat analog, including the technology of cultured meat production, can solve the problems of traditional meat production, such as inadequate breeding environment, wastewater, methane gas generation, and animal ethics issues [4]. Therefore, this study was conducted to investigate the history of meat analog materials, current technologies, and future market prospects, and to provide the negative effects and solutions of the growth of the meat analog market in the livestock industry.

## CURRENT MARKET STATUS OF MEAT ANALOG IN THE U.S

### Market condition of meat analog in the U.S

Alternative foods have entered major markets due to the spread of animal welfare debates and positive perceptions of vegetables, thus showing rapid growth rates. In addition, Market Research Future, a market survey, analyzed that after the start of the Coronavirus disease 2019 (COVID-19) pandemic, many consumers had an opportunity to choose vegetable protein over animal protein [5]. The U.S. market for alternative foods increased 11.4% year-over-year to $4.98 billion in 2019, and the average annual growth rate of 13.4% over the past three years (2017 through 2019) has increased due to increased interest in health, environment, and animal welfare issues and the spread of positive awareness of vegetables [6].

The growth rate of overall food sales in 2019 was 2.4%, but the growth rate of alternative food sales was 11.3%, showing a five-fold increase. In particular, vegetable milk accounted for 14% of the total milk category in 2019, while vegetable butter and creamer accounted for 6% and 5%, respectively [7]. The high price of alternative foods is still an obstacle to the growth of alternative foods, but the alternative food market is expected to grow 13.1% annually over the next five years, reaching $9.22 billion by 2024 [8].

Under U.S. federal law, the term for alternative food is de fined in “special dietary and nutritional additives” as subpart D of part 172 in subchapter B of title 21 in the Code of Federal Regulations (CFR). It can be organized into “Edible plant-base~,” “Soy-based~,” and “Plant protein products” [9].

### Alternative food certification system and labeling in the U.S

Products currently being released by alternative food companies, such as Impossible Foods, Beyond Food, and JUST Food, are labeled as plant-based alternative meat products. On the front of the product, the words “~made from plants” and “plant-based~” are used together with terms such as “meatballs” and “burgers.” Certification authorities related to this include Plant Based Foods Association, BeVeg, the American Vegetarian Association, and Vegan Action [8]. Plant Based Foods Association launched the first “Certified Plant Based Seal” program in the U.S. in 2018 and appointed the National Sanitation Foundation International as a certification authority [10].

The Food and Drug Administration (FDA) has a flexible approach that allows for the use of terms such as “milk,” “cheese,” and “meat” when words such as “vegan” and “plant-based” are displayed. However, sanctions can be imposed if they confuse consumers or if they are false or misleading, i.e., the labels are intended to clearly communicate the properties of alternative foods [11].

The Plant Based Foods Association establishes voluntary labeling standards that reflect federal and state requirements as follows to increase the consistency of labeling for alternative foods. Starting with vegetable milk in 2018, labeling standards for alternative meat in 2019 and vegetable yogurt in May 2020 were announced [6,8,12,13].

### Plant-based milk labeling in the U.S

The Plant Based Foods Association recognizes a product as vegetable milk when the proportion of solids derived from one or more combinations of specialized ingredients is not less than 2% of the final weight or volume of the final product. The specialized ingredient refers to a raw material for preparing vegetable milk, such as nuts, beans, grains, and seeds, and the milk may have more than one specialized ingredient [14].

The specialized ingredient may be indicated by one word (e.g., Almondmilk) or two words (e.g., Almond milk) that includes the characteristic ingredient with the word “milk,” and may be hyphenated (e.g., Almond-Cashew Milk) if the product is made of two or more specialized ingredients [15]. Plant-based milk (including or excluding hyphens) may be marked if the specialized ingredients are clearly marked, and all vegetable milk shall be clearly marked as “dairy-free” or “non-dairy” in a conspicuous position on the main surface [8].

### Plant-based meat labeling in the U.S

The Plant Based Foods Association defines alternative meat as food produced from vegetable ingredients that may have the texture, flavor, appearance, and other characteristics of actual meat but do not contain animal ingredients [13].

Alternative meat products may be labeled with terms such as “meat,” “burger,” “sausage,” “chicken,” “pork,” and “ham” if the product is clearly described as plant-based. Labeling of product types can be displayed according to the shape or form, such as nuggets, tenders, burgers, patties, etc., and words describing the flavor, texture, purpose, and form can also be used on the label [16]. The label shall use one or more of the following terms or phrases to accurately indicate that it is plant-based in the most prominent position on the main surface (i.e., Plant-based, Vegan, Meatless, Meat-Free, Veggie, Vegetarian, Made from Plants, or Veggie-based) [8,16].

### Agreement on alternative foods in the U.S

In the U.S., the debate over alternative foods related to animal products (alternative meat, alternative cheese, alternative yogurt, etc.) is intensifying, and various bills are being proposed in each state [17,18].

In particular, the debate over alternative meat products has spread to several states in the U.S., and legislation is currently being proposed to prevent plant-based or cell culture products (cultured meat) from being classified as “meat” or “beef” in Mississippi, Oklahoma, Washington, Louisiana, and South Carolina [19]. There are 13 states (Alabama, Arkansas, Georgia, Kentucky, Louisiana, Mississippi, Missouri, Montana, North Dakota, Oklahoma, South Carolina, South Dakota, and Wyoming) that have passed the ban on the use of the meat term, and more states (Arizona, Colorado, Hawaii, Illinois, Indiana, Iowa, Kansas, Maryland, Michigan, Nebraska, New Mexico, Tennessee, Texas, Vermont, Virginia, Washington, and Wisconsin) have proposed the bill but have not passed it [20]. The “Real Marketing Edible Artifacts Truthfully Act of 2019” or “Real MEAT Act of 2019” was introduced in 2019 as a law requiring products similar to beef but not derived from cattle to be labeled with the word “imitation” [21].

The issues currently being discussed on labeling by states in the U.S. (for states where the law has been passed) are summarized in Table 1. On the other hand, the Good Food Institute and plant-based food retail companies, led by the Plant-Based Food Association (PBFA), have had steadily increasing sales of plant-based alternative foods every year, and they continue to file petitions asserting that the present notation is not confusing to consumers [22]. In addition, based on the Harvard Animal Law & Policy, the Tofurkey organization hopes to use meat and beef on the label of the product. Turtle Island Foods (Tofurkey brand) is currently suing federal courts for a ban on the use of “meat” terms, claiming it violated its rights to Amendments 1 and 14 with a nonprofit advocacy group [23].

In the case of California, the Northern District Court ruled in August 2020 that the term “butter” should be used for vegetable dairy products. The Food Safety Inspection Bureau (FSIS) under the USDA and the FDA have signed an official agreement on measures to regulate cultured meat. The USDA and FDA agreed that meat and poultry culture is meat and poultry and agreed to manage meat and poultry culture under the Federal Food and Drug Cosmetics Act (FDCA), Federal Meat Inspection Act (FMIA), and Poultry Inspection Act (PPIA) [24].

The FDA regulates and supervises the production of prod ucts in the cell culture process, including cell collection, cell banking, and cell growth and differentiation, and has jurisdiction over products containing not more than 3% raw meat or less than 2% cooked meat or poultry products. The USDA regulates and supervises post-production processes (processing, packaging, and labeling) including monitoring and labeling, but there are still arguments for and against marketing and advertising them. FSIS announced that it would address cultured meat issues in the advance notice of proposed rulemaking (ANPR) (Rules on Labeling of Cellular Cultivation Meat) enacted in September 2021, without adding new items to food standards and labeling policies for alternative foods, and that ANPR will manage labels for plant foods as well as cell-derived cultured meat. In addition, the FDA Center for Food Safety and Applied Nutrition (CFSAN) said it will publish guidelines on new dietary ingredients, labeling of vegetable milk alternatives, and labeling of animal foods by January 2023 [9]. There is a continuing debate in the U.S. over the labeling of alternative meat, including vegetable and cultured meat. In particular, Louisiana (where the meat term ban was passed) appealed, claiming that the Good Food Institute and The Animal Defense Fund violated the First Amendment. In the March 28, 2022 ruling, a Louisiana judge accepted an appeal and allowed consumers to use meat labels (using vegan and plant-based) on the premise that they are not using misleading labels; therefore, it remains to be seen how the lawsuits filed in each state will proceed [25,26].

## CURRENT MARKET STATUS OF MEAT ANALOG IN EU

### Market condition of meat analog in the European Union

In Europe, plant-based alternative foods, plant-based meat, cultured meat, and milk products are listed according to the naming regulations. The issues currently being discussed on labeling are summarized in Table 1. The EU Court of Justice ruled against the use of milk or cheese names in vegetable dairy products. In principle, the name milk is defined as produced through mammary secretion of animals (853/2004/EC), and words such as “milk,” “cheese,” and “cream” can be used in products derived from mammary secretions. Therefore, the ruling stated that the name “milk” or “cheese” can only be used for the sale and advertisement of dairy products based on animal ingredients. In other words, the name of the dairy product cannot be used even if it is combined with words such as “vegan” and “vegetability”. However, as an exception, the EU allows dairy names to be used only for “the exact nature of which is clear from traditional use” (coconut milk, peanut butter, etc.) (Commission Decision 2010/791, 1234/2007/EC). However, amendment 171 (discussed below), which restricts the use of dairy terms such as “buttery” and “creamy” for products that do not contain dairy products, was rejected (2020.10) [27].

Regarding the name of meat, the Member of the European Parliament (MEP) of France submitted an amendment (2019.4) to the Commission’s proposal for reform of the Common Agricultural Policy (COM/2018/0394). The key point of this amendment is to prohibit the use of meat-related names (steak, sausage, escalope, burgers, etc.) in labels and food descriptions of vegetarian or vegan foods. In response, The Vegan Society, a British non-profit vegan organization, sent an official letter to the EU saying that the bill violates EU laws on accurate labeling and violates consumers’ right to receive information. This amendment was rejected by the European Parliament (October 23, 2020), which prohibits the use of names insinuating meat in plant-based meat alternative foods. The rationale for this is that certain meat names for beef, chicken, and pork are protected, but names representing the form and composition of “meat products” are not protected. Therefore, it is currently possible to use meat names, such as “sausages” and “burgers,” in plant-based foods within the EU (Regulation 1308/2013/EC) [28].

### Novel Food Regulation in EU

The European Union has established a new food regulation (2015/2283/EU) which defines “food that has never been consumed in the EU market due to new raw materials or previously unused raw materials or previously unused production processes before 15 May 1997.” Noble Food was first introduced on May 15, 1997, and it is a system that evaluates and approves the safety of new foods and ingredients excluding food additives and new food production methods to protect consumers. Noble Food refers to food or food ingredients that have no food history in Europe and require evaluation for safety. The process of introducing Noble Food in the EU that were not previously consumed there is done to confirm and evaluate its safety before sale. For example, insect food, tooth seed, baobab, krill oil, and cultured meat are managed within the category of Noble Food, and in particular, alternative foods are defined as follows: “Food separated or produced by cell culture or tissue culture extracted from animals, plants, microorganisms, fungi, and algae.” In accordance with the Noble Food Regulations, the EU operates a system managed by the European Commission for registration and approval of new foods [29].

Noble Food follows the following principles: i) Ensuring consumer safety, ii) Use correct labeling that does not confuse consumers, iii) If Noble Food replaces other foods, there should be no nutritional deficiencies, iv) In the case of cell culture meat, it is subject to GMO Food and Feed Regulations and New Food Regulations for food permission, and v) Cell culture meat using GMOs, such as induced pluripotent stem cells, follows the GMO Food and Feed Regulations, and all other cell culture meat follows the new food regulations. Meanwhile, the European Parliament has supported research and development projects for cultured meat since 2019, which can be seen as acknowledging that the cell culture industry will be an important food source in the future. According to market researcher Grand View Research, the size of the European alternative food market was estimated to be about 1.7 trillion Korean won in 2019 and will continue to grow at an average annual rate of 7.3% by 2025, especially in the UK, Germany, France, Italy, and the Netherlands. The growing size of the alternative food market in Europe coincides with a decrease in meat consumption, and more than 40% of the respondents have already stopped or reduced meat consumption in 11 European countries (Greece, Netherlands, Germany, Lithuania, Belgium, Spain, Slovakia, Slovenia, Austria, Italy, and Portugal). The global alternative food market size was approximately $4.7 billion in 2019, and it was found to have a size of $6.1 billion (12.9%) in the UK, $2.60 billion (5.5%) in Germany, and $2.0.3 billion (4.3%) in Italy.

Quorn, a leading British alternative food manufacturer, produces alternative foods based on mycoprotein, and sells products in various forms, such as chicken breasts, steaks, and sausages, using fungal proteins obtained through microbial fermentation. Founded in the 1990s, OATLY in Sweden produces oat-based vegetable milk alternatives. In particular, OATLY claims that consuming one liter of the product reduces greenhouse gas emissions by 80%, land use by 79%, and energy consumption by 60%.

## CURRENT MARKET STATUS OF MEAT ANALOG IN SOUTH KOREA

### Market condition of meat analog in South Korea

As of 2019, in South Korea, the marketing of meat analog as a plant-based alternative meat was $17.4 million [30], which was found to be the world’s 38th largest industry, indicating that it is in the stage of market formation. In 2021, it entered the period of market introduction with $11.1 billion (11% compound annual growth rate). Although there is no legal definition of the term “alternative” in South Korea, the use of the “alternative” term has continued with similar standard terms by establishing new types. Particularly, new types of foods or terms have also continued when “alternative foods” are developed according to the characteristics of the resources (plant and animal properties). In 2025, the market of plant-based alternative meat in South Korea is expected to grow to $22.6 million, which is predicted to increase 29.7% from 2020. Soybean accounts for 62% of the total market (being the largest portion of the market depends on raw source types), followed by vegetable protein, and grains.

The research on cultured meat in South Korea is at an initial stage and is evaluated to be about 4 to 5 years behind the technology level compared to that of overseas countries. Notably, an increasing number of companies are developing cultured meat. In addition, it is expected to be a market with potential growth as research and funding by major companies and venture companies (CJ CheilJedang, Dae Sang, SpaceF, Cell meat, DaNAgreen, and SeaWith) have been formed. It has been suggested that it would be better to recognize cultured meat as a new food and that it should be classified as a new industry rather than a livestock industry. In addition, it can be suggested that it is favorable to introduce new terms, such as “cell-derived protein foods,” rather than adding “meat.”

## CURRENT MARKET STATUS OF MEAT ANALOG IN JAPAN

### Market condition of meat analog in Japan

The Japanese government has established a policy to focus on developing alternative meat-related food techniques to improve the global environment, food problems, and national health, and the size of Japan’s alternative meat market is expected to expand more than 40 times in 10 years to 30.2 billion yen in 2030 [31]. Fuji Oil, a food material processing company, is operating a new plant-derived meat factory to accelerate sales to restaurants in addition to consumer homes, while Marudai Foods and Otsuka Foods are selling hamburgers and other products for restaurants [31]. Japan’s AEON company has started selling plant-based meat in the form of retail meat cuts. Its texture and taste have improved, and meat-type products have appeared in meat corners, improving consumer awareness. In January 2020, Nissin Food and Nippon Ham launched a cell agricultural research group to promote industrialization of cultured meat.

### Definition and labeling of alternative foods in Japan

Replacement meat is classified into cultured meat and plant-derived meat, and cultured meat can be divided into two types: i) Something that can eat the cells themselves and ii) Something that is made by extracting the desired protein by increasing yeast cells. Article 2-2 of the Japanese Agricultural Standards Act (JAS Act) states, “Agricultural products, forest products, animal products, and fisheries products and goods manufactured or processed as raw materials or ingredients (excluding those listed in the preceding item) are prescribed by the Cabinet Order” [32]. It can also be read as animal products or livestock products manufactured or processed with raw materials or ingredients. It is necessary to wait for future discussions on the interpretation of the law, but it is also necessary to determine the position of “culture” as a new method of food production. Plant-derived meat is a processed food that has the same texture as livestock meat by extracting proteins from raw materials, such as beans and vegetables, and then heating, cooling, and pressing.

Plant-derived meat is classified as “other processed foods” according to the Classification of Processed Foods in Japan, and raw material labeling is important. On February 24, 2022, “Textured Soy Protein Products” was established in the JAS 0019 and added to the Minister of Agriculture, Forestry and Fisheries list, indicating certification technology standards and inspection methods, which defined the requirements for “soy meat products” and “prepared soy meat products.” According to Food Labeling Standards, the label on retail processed foods must contain “general names,” and general names cannot refer to ingredients not included in processed foods. In other words, it is impossible to label foods made of soybeans as “alternative meat,” and companies are currently labeling foods such as “soybean-based processed foods.” The description that the product is not meat should be entered in a location that is readily visible on containers or packaging, and the common names such as “meat” and “egg” should not be used in the raw material names of vegetable meat. The use of terms such as “soy-based meat” or “oat milk” on vegetable labels is permitted if the label states that the product is not animal meat (e.g., No meat, soybeans used as raw material, or raw). Exemption provisions such as “100% vegetability” are also available, but all ingredients used must be derived from plants.

Differences in food classification and food labeling (label ing) contents for each country (Table 1) was investigated. Unlike other countries, the U.S. has a very large debate about labeling alternative foods. Each state clarifies the definition of “meat” to protect the livestock industry and prohibiting the use of the term “meat” for alternative meat and cultured meat and allowing it to be used with vegetable words (such as “vege,” “plant-based,” etc.) are different from state to state.

In China and Japan, when consumers purchase alterna tive foods, the raw material name can be clearly stated, or if used with plant-related words, such as “vege” and “plant-based,” the alternative food classification is classified according to the existing food classification system.

In addition, Australia conducts vegan certification for meat replacement foods in Vegan Australia, and companies use logos for one year after an annual renewal as well as vegetable replacement foods mixed with livestock foods in accordance with the Food Standards Regulations and Food Standards Code (Section 1.1.1—13(4)). New Zealand also uses plant-based alternatives, and Singapore has approved the world’s first cell-cultured food through the establishment of guidelines for new food safety assessments, which should be characterized by appropriate terms such as “mock,” “cultured,” “imitation,” “plant-based,” and “mat-baked” if only plant-based.

Alternative foods currently distributed in Korea (exclud ing cultured meat) are classified by the definition of each food type in the Food and Drug Administration’s food industry, and terms such as “hamburger patties,” “meat,” “nuggets,” and “steaks” are labeled on the cover of the product. However, in Korea, the issue of terminology labeling is currently being raised in the domestic livestock industry (like the United States; therefore, it is time for experts from all walks of life, livestock farmers, and consumers to reach an agreement.

## REGULATION FOR THE INDUSTRIALIZATION OF MEAT ANALOG

### Regulation for the industrialization of meat analog in the U.S

Since plant-based, insect-based, and microbial-based meat analog are mostly manufactured using approved ingredients as foods, the related laws were excluded, and the law or regulation of representative countries related to cultured meat was investigated. The results of a survey of the U.S. regulation for the industrialization of cultured meat are as follows:

In the U.S., the responsibility for food safety exists with the Food and Drug Administration (FDA) and the United States Department of Agriculture-Food Safety and Inspection Service (USDA-FSIS) to specify and jointly regulate some of the regulations on cultured meat, which began on March 7, 2019. Considering the specific role of each organization in regulating and inspecting food and agricultural products, the USDA and FDA have decided to take joint and partitioned responsibilities in the regulation of cultured meat, as shown in Table 1. The FDA did not provide specific requirements for tissue collection and cell culture but said cultured meat products produced from cultured animal cells would undergo a thorough market transfer process, record and facility inspection, facility registration, and compliance with FDA current good manufacturing practices (cGMP). In conducting inspections and other supervisory activities, the FDA will take appropriate measures against the institution if the inspection results confirm nonconformity by utilizing the consultation results of the market transfer phase and the thorough evaluation of production records maintained and managed by the facility. After the process of “harvesting” cells or tissues, jurisdiction is changed from the FDA to the USDA-FSIS. Organizations that harvest cells must meet USDA-FSIS regulatory requirements, including: i) The inspection application must be submitted, ii) The facility must meet the standard performance, iii) Installation hygiene, and iv) hazard analysis critical control points (HACCP).

The USDA-FSIS inspector reviews records generated dur ing cell culture and verifies compliance with applicable USDA-FSIS regulatory requirements during product processing, packaging, and labeling to ensure that the product is safe, complete, beneficial, and properly labeled. Upon completion of the cell collection and processing tests of the USDA-FSIS, a USDA test mark is received, and the quality of cultured meat products is guaranteed to be genuine and consistent. These agencies agreed that cultured meat products are meat within the definition specified by the FMIA. However, in January 2020, 12 states passed laws restricting the use of terms such as “meat” in cultured meat products, but a clear labeling system created by FSIS has been reported to prohibit state laws restricting the term “meat” for cultured meat [33]. According to the Good Food Research Institute [34], a non-profit organization in the U.S. that promotes the alternative meat industry, cell culture contains ingredients widely used in the food industry, such as salt, sugar, and amino acids, and the organization aims to document their safety. Article 402 of the Federal Food, Drug, and Cosmetic Act (FD&C) considers food additives to be poor if they contain unsafe food additives within the meaning of Article 409 of the Act and additives are considered poor even if they are manufactured, packaged, or stored under unhealthy conditions. On May 31, 2021, Texas approved a bill banning companies producing food from insect, plant, or cell culture, such as alternative meat and cell culture, from using the term “meat” in product labels in the future.

### Regulation for the industrialization of meat analog in EU

The EU regulations related to the industrialization of cultured meat were investigated as follows: In the EU, cultured meat is regulated by the New Food Regulations (NFR, Novel Foods Regulation 2015/2283) because foods composed, separated, or produced by cell and tissue culture from animals, plants, microorganisms, fungi, or algae are considered one of the new food categories listed in this Regulation. The EU’s NFR stipulates that the issue of labeling cultured meat as “meat” should not be different from the food in a way that is less nutritionally beneficial to consumers if there is a new food to replace other food. This definition focuses on nutritional value and may be advantageous for cultured meat because it is low in fat [33]. The EU requires a verification process to verify the safety of cultured meat producers before introducing their products to the market, and they must obtain prior market approval, including a safety assessment conducted by the European Food Safety Authority (EFSA). Food companies must pass certain approval procedures under NFR 2015/2283. An online application must be submitted, including scientific evidence proving that the product poses no risk to human health and safety, and a proposal for specific label requirements for a particular product must be included. Details that the application must contain are as follows:

- The product may be subjected to a safety assessment by EFSA to determine its impact on human health in accordance with section 11 of NFR 23.
- According to the EU’s Novel Foods Regulation, applications for approval of cultured meat are made through an e-submission system operated by the European Commission and the minimum requirements for approval consist of information on the product ID, production processing, configuration data and specifications, proposed uses, and expected product intake.
- Upon receipt of a new food application, the European Commission requests a safety comment from EFSA, which evaluates whether this new food has the same safety as the similar food present in the EU market.
- Within 7 months of receiving positive feedback on safety, the European Commission shall publish the Enforcement Act, and as a result, new food approved shall be included in the Union List.

### Regulation for the industrialization of meat analog in Singapore, Israel, Australia, and New Zealand

The Singapore regulations related to the industrialization of cultured meat were investigated as follows:

Singapore has provided detailed “Guidelines on New Food Safety Assessment” to be considered when approving new food applications by its competent authority, the Singapore Food Agency (SFA). In the case of *in vitro* meat has been stipulated, such as in Table 2 below.

The Israel regulations related to the industrialization of cultured meat were investigated as follows:

Israel has a pre-market approval process as described in the NFR Framework under Article 18 of the Public Health Food Protection Act. New foods in this process are divided into three categories.

The necessary safety assessments are modeled according to the EU assessments, and the Israeli regulations accept safety assessments from EU, US, Canada, Japan, Australia, and New Zealand organizations.

The Australia and New Zealand regulations related to the industrialization of cultured meat were investigated as follows:

Food Standards Australia and New Zealand (FSANZ) stated that the Food Standards Code stipulates how foods produced by new technologies, such as cultured meat, should be handled.

Cultured meat shall comply with the Standard Code and shall be approved at the market transfer stage in accordance with the new food regulations. It stipulates that new foods, such as cultured meat, require evaluation for public health and safety considerations, including i) Potential side effects in the human body, ii) Structure and components of food, iii) Food manufacturing process, iv) Patterns and levels of food consumption, and v) Other related matters. Post-market monitoring is important to ensure that cultured meat is not unbalanced or causes side effects. Given that cultured meat has not yet been marketed, post-market monitoring may be useful for learning quickly in the event of negative situations. The food companies should establish services so that consumers can report any side effects from the meat, and in particular, research on cultured meat consumers should be conducted to measure consumption levels and nutritional levels. In addition, if food authorities desire independent consumer information about cultured meat, they should establish their own infrastructure lines or websites that allow consumers to provide feedback directly.

## STATUS OF GLOBAL ALTERNATIVE FOOD RESEARCH AND INDUSTRIALIZATION

The global industrialization status of alternative food technologies was performed to launch alternative food products with forming meat products, vegetable protein drinks, seafood, insect burgers, protein bar products, and cultured meat, as summarized by this study in Table 2. Moreover, many companies in Korea launched alternative foods, which are made to use plant-based protein, such as some vegetables, wheat, beans, grains, and fat extracted from edible insects, such as *Tenebrio molitor* and crickets (Table 3). The research and industrialization relevant to alternative food have consistently increased and some processes have led to obtaining meat-like characteristics, such as for alternative food development, and cell growth for cultured meat (Table 4).

Leghemoglobin is usually used as a safety flavor catalyst in plant-based meat. A leghemoglobin gene can be extracted from soybean root lump through fermentation of the *Pichia pastoris* Bg11 strain yeast, which leads to the production of high concentrations of heme [35]. The yeast used are lysed by mechanical shearing after the fermentation process, the solution containing heme is then separated by centrifugation and microfiltration, and the separated heme concentration is standardized to a final concentration of 6% to 9% soy leghemoglobin protein [11]. The Impossible Burger is designed to obtain a meat texture using representative protein from wheat and potato, coconut oil for meat juice, leghemoglobin to improve the flavor profile to resemble meat, and a colorant with red-colored properties to resemble meat when cooked.

However, there are concerns about the health and envi ronmental influences of the Impossible Burger related to the safety of the genetically modified organisms employed by use of their leghemoglobin in the initial development stage. For this reason, the Impossible Burger is required to be studied in terms of a safety assessment to be accepted by the consumer. Jin et al [36] found no evidence to suggest that food containing soy leghemoglobin protein and minor content from yeast showed any risk of allergenicity or toxicity, and they were quickly digested by pepsin and a pH 2 environment. The safety of leghemoglobin, which provides nutrition, and the flavor and aroma were measured using the bacterial reverse mutation and *in vitro* chromosome aberration assays relevant to genotoxicity [37]. The leghemoglobin in this study was nonmutagenic and nonclastogenic under these assays. In addition, the administration of leghemoglobin at 750 mg/kg/d in Sprague Dawley rats did not cause mortality and adverse clinical reactions [37].

Plant-based meat is generally made to fabricate vegetable ingredients that can be used for flavoring, simulating meat texture and providing nutritional and functional properties. Alternative meat products developed using leghemoglobin extracted from soybean root lump can supply flavor and simulate a texture that is similar to that of conventional meat products [38,39]. Some authors developed the method for producing meat from soybean protein mixtures with excellent nutritional and functional properties and meat-like microfiber structural forms [40], as well as alternative sausage made from concentrated soybean protein, textured vegetable proteins (TVP), and textured soybean protein (TSP) extracted from soybeans, mung beans, red beans and peas [41].

Mycoprotein, which is a fungal protein with a high-pro tein mass through fermentation of fungi, is more nutritious, has a meat-like texture and various functional properties, and is a promising protein source to alternative meat from plants or animals. Previous studies suggest that the intake of mycoprotein can improve the lipid profile, reduce energy intake, and stimulate muscle protein synthesis [42]. Mycoprotein has low cholesterol, a low lipid content in saturated fatty acids, and high polyunsaturated fatty acids [43,44]. Particularly, mycoprotein is a high biological value protein that has high dietary fiber, some minerals such as iron, zinc, selenium, and phosphorus, and vitamins.

Mycoproteins have been produced by numerous strains, such as *Rhizopus oryzae*, *Paradendryphiella salina*, *Neurospora intermedia*, and *Aspergillus oryzae*, via submerged fermentation, solid-state fermentation, and surface culture [42,45,46]. Submerged fermentation is used to produce mycoprotein biomass from brown giant algae and seaweed waste with the marine fungus *Paradendryphiella salina* [45,47]. Solid-state fermentation is used to produce mycoprotein through the solid fermentation process from fruit waste (watermelon, cucumber, orange, banana, and pineapple waste) and agricultural waste (brewer-spent grain, grape bagasse, and stale bread) with *Aspergillus Niger*, *Neurospora intermedia*, *Rhizopus oryzae*, *Agaricus blazei*, A*uricularia fuscosuccinea*, and *Pleurotus albidus* [48,49]. The surface culture method is applied to culture various edible fungi strains in pea processed byproducts for mycoprotein production [50,51]. Solid-state fermentation has been widely performed to produce traditional fermented foods in different scale instruments. The difference between submerged fermentation and solid-state fermentation is that a low-moisture solid substrate is used in the latter, while a high-moisture liquid medium is used in the former to cultivate microorganisms [42]. In the production of mycoprotein, glucose, water, and *Fusarium venenatum* are added into the fermentation tank, and minerals such as calcium, magnesium, and sodium phosphate are added when culturing begins. In addition, air and ammonia are injected to supply oxygen and nitrogen that can produce protein and help respiration, which leads to the production of protein solids after 5 to 6 h. The proteins are produced through the degradation of hexane in protein solids, centrifugation, and the drying process [52]. The Quorn fermentation process involves the following steps: i) fermentation to grow the organism; ii) RNA reduction to meet specifications; iii) centrifugation to separate solids and liquid; and iv) chiller to harvest mycoprotein paste [20]. The mycoprotein obtained from the fungus *Fusarium venenatum* A3/5 (ATCC PTA-2684) has been used to produce the meat alternative product Quorn [53]. Furthermore, the retentate fraction, which was an unexploited co-product from the Quorn fermentation process, showed an entanglement of mycelial aggregates and filaments that is similar to the microstructure of Quorn products and has a meat-like texture [54,55].

Yeast has been applied to enhance the red color and provide lipids and antioxidants for developing alternative sources [56]. Lipids and carotenoids, which are responsible for imparting a red color and supplying fatty acids appliable to the food industry, are compounds obtained from the *Rhodosporidium toruloides* yeast using various substrates [56]. Lee et al [57] also found that the use of the oleaginous yeast *Rhodosporidium toruloides* increased the carotenoid concentration from 1.9 to 2.9 μg/mg and the fatty acid yield from 0.07 to 0.09 mg/mg through the glycerol and succinic acid metabolic pathways.

Edible insects are developed into high quality alternative protein sources; 1,400 species of edible insects are known, which are used in industrial applications as food and animal feed sources [58]. The nutritional value of edible insects includes rich protein, lipids, fatty acids, minerals, vitamins, and chitin. Xia et al [59] investigated the protein content of *Clanis bilineata* (Lepidoptera) and found that it ranged from 400 to 750 g/kg dry weight. Kouřimská and Adámková [60] showed the protein content of insects varies from 20% to 76% of dry matter depending on the species and growth stage of the insect. Lipids and fatty acids are the second largest content of insects, and they vary from 10% to 60% of the dry matter in edible insects. These contents were higher in the larval stage than in adult insects [61].

Various extraction methods in edible insects, such as en zymatic hydrolysis and sonication, have been applied to improve the techno-functional properties. Acquisition of protein in edible grasshopper (*Schistocerca gregaria*) and honeybee brood (*Apis mellifera*) was performed via processes, such as defatting, alkaline, and sonication-assisted extractions, resulting in the production of protein enriched powder [62]. The protein fractions in grasshopper and honeybee brood showed high foaming (74.1% in alkaline extraction and 55.5% in sonication-assisted extraction) and emulsifying abilities (100%). In addition, protein extraction changed the molecular characteristics, leading to improve functionality. Moreover, chitin, which is responsible for indigestion and has no nutritional value, was removed [62]. Edible insect proteins from migratory locusts were degraded by various proteases (Alcalase, Neutrase, Flavourzyme, Papain) or the enzyme complex depending on the enzyme-substrate ratio, heat pre-treatment, and hydrolysis time [63]. This result showed that the use of the enzyme complex was effective in migratory locust protein hydrolysis and resulted in the improvement of techno-functional properties as functional ingredients. The migratory locust protein hydrolysate improved in solubility (pH 3 to 9), emulsifying activity (pH 5 to 7), and foamability (pH 3 to 5) as compared to that of the non-hydrolyzed migratory locust protein [63]. Lee et al [64] also studied that *Protaetia brevitarsis* larvae hydrolyzed by five proteases (alcalase, bromelain, flavourzyme, neutrase, and papain) showed differences in hydrolysis degree, antioxidant activities, and peroxidation inhibition depending on these enzymes. Among these enzymes, the use of alcalase was higher in the production efficiency of the low molecular peptide (<3 kDa) with high antioxidant activities than that of the others. However, although edible insects are superior protein sources to produce alternative protein, some concerns are known about the safety aspect, such as allergenicity, toxicity, and food neophobia of eating insects. For these hurdles, numerous studies have been conducted to confirm the risk of using edible insects, and they confirmed that there were no problems concerning the acquisition process of protein. Enzymatic hydrolysis reduced allergenicity, such as the solubility of the IgE-binding protein [65,66].

The edible insects and insect-based food products were lower in all contaminants, such as organic and metal contaminants (heavy metals, DDT, and dioxin compounds), than other common animal products [67]. Cricket powder increased the growth of the probiotic bacterium *Bifidobacterium animalis* and also reduced plasma tumor necrosis factor-alpha (TNF-α), which improved gut health and reduced inflammation [68]. Frankfurters made with 40% pork meat and 10% yellow mealworm were higher in protein content, ash, pH, and yellowness than that of the 50% pork ham, and were similar overall to the acceptability of the regular control frankfurters [69].

Cultured meat or *in vitro* meat is the most representative alternative meat produced by cells obtained from animal tissues. Unlike conventional meat, the production of cultured meat does not require raising livestock and can be defined as cell agriculture that produces animal protein. Moreover, the reproducibility of the meat flavor is excellent because it is more similar to conventional meat as compared to plant-based meat and edible insects. Many studies have been conducted to develop the cultured meat industry into a wide field, such as cell types, serum-free media, ingredients, technologies, and materials [4,70–72].

Animal muscle tissues have been widely used to obtain stem cells for cultured meat, and the stem cells can successfully produce muscle fibers via differentiation into myocytes and myotubes or fat cells [73]. Lyu et al [74] investigated the composition of a subpopulation that differs in myogenic potential in bovine satellite cells with single-cell RNA sequencing using the 10× Genomics platform. Clustering the transcriptomes of 19,096 cultured bovine satellite cells revealed 15 cell clusters that had similar gene expression patterns. In addition, myoblast determination protein 1 (MYOD1), myogenic factor 5 (MYF5), and desmin are markers of myoblasts, which are activated and proliferating satellite cells, and were seen in the myoblast subsets in clusters 1, 2, 3, and 12. The remaining two clusters were dominant in platelet-derived growth factor receptor-α (PDFGR-α), which is a marker of fibro-adipogenic cells [74].

The study observed the metabolism of C2C12 myoblasts and myotubes in serum-free medium (B27, AIM-V) compared to normal culture media with serum by observing the cell morphology and viability of myoblasts, as well as myotube formation via myogenic differentiation, for 7 days [75]. Metabolic differences are found to be more dependent on the state of the cell than on the effect of the medium. In addition, the C2C12 myotubes cultured in serum and B27 have a prominent glycolytic and oxidative metabolism, respectively, which is observed in muscle types (fast and slow) identified by major histocompatibility complex (MHC) immunostaining. The metabolic profiles (phosphorylated metabolites and tricarboxylic acid intermediates) in the AIM-V culture were similar compared to the serum culture [75]. Other studies developed serum-free media using silkworm fibroin as an alternative for fetal bovine serum (FBS) in animal cell culture [76]. The study found that the cell viability was higher in culture media with fibroin than that of culture media with FBS and showed differences depending on the silkworm varieties. The use of mushroom concentrates and bovine satellite cell culture media produced patties using the bottom spray under the fluidized coating condition. The patty was cultured meat obtained prior to the muscle fiber formation step, which produced a texture and taste like that of meat [77].

An effective four cytokine combination promoted the long-term proliferation of porcine muscle stem cells (6.31× 10^7^-fold cell increase) and reduced the need for FBS in long-term culture to 5%. The four cytokines identified were Long arginine 3-insulin-like growth factor -1 (LR3-IGF-1), human recombinant platelet-derived growth factor (PDGF-BB), basic fibroblast growth factor (bFGF), and epidermal growth factor (EGF) that activate via the PI3K/Akt/mTOR and MEK/ERK signaling pathways, and they may allow the industrialized development of cultured meat [78]. Other studies observed that cell proliferation and stemness maintenance on porcine muscle stem cell depends on the density, which can enable production of large-scale cultured meat [79]. Activating yes-associated protein (YAP) promoted the proliferation and differentiation of potential porcine muscle stem cells as compared with control cells. Moreover, YAP with phosphorylation sites deactivated elevated cell proliferation and stemness maintenance under a high cell density, which generally impairs cell proliferation and differentiation [79]. The decellularized spinach as an edible scaffold showed a 99% survival rate and differentiation of bovine satellite cells, which was similar to the control on gelatin-coated glass [80]. In addition, structuring technologies or instruments have been developed to form the structure of meat analog. Three-dimensional cultured meat technology applies biomaterials using fat cells derived from cow muscles to 3D bio-printing technology [81]. Gelatin methacryloyl (GelMa)-based bioink with cells provided a scaffold fabricated to encourage the stable adhesion and high proliferation of cells, which suggests the possibility of using this technology in cultured meat production [82]. Furthermore, various materials, such as microcarriers, seaweed, double bridge, and a casting tray have been studied for manufacturing a 3D cell culture support for large scale cultured meat production. Therefore, numerous strategies, such as serum-free media, cytokines, protein-relevant specific pathways, and edible ingredients have been continually attempted to produce large-scale cultured meat.

## CONTROVERSY OF INDUSTRIALIZATION FOR MEAT ANALOG

Despite the great enthusiasm for cultured meat in capital markets, there are still major challenges to the maintenance of sustained and stable development in this field [83]. The low productivity caused by technological bottlenecks is the key factor in restricting the commercialization of cultured meat and regulatory system improvement [83]. The production of cell-based cultured meat is a complex technological process that integrates multiple technical fields, such as tissue sampling, cell culture and fermentation, 3D printing, and meat processing. Therefore, the imitation of meat, a highly complex product with a well-appreciated, distinctive flavor and texture, remains a technological challenge [2,84]. In addition, unlike in the beginning of the product’s launch, there is continuous news that sales of plant-based meat alternatives in the U.S. are gradually decreasing. The main reason is that the taste and quality of vegetable substitute meat do not meet the quality of traditional meat. For plant-based meat alternatives to replace traditional animal products, they must have a comparative advantage over traditional animal products in terms of flavor, quality, nutrients, or price to survive in the market. One of the main purposes of developing cultured meat is to protect the environment and reduce greenhouse gas emissions by replacing the traditional livestock industry; however, the stem cells needed to manufacture cultured meat must be obtained from livestock. Therefore, cultured meat cannot completely exclude the connection with the livestock industry, and there is a limitation in that the use of animals cannot be completely excluded. In summary of the current status of meat analog, industrialization has not grown as much as expected in the plant-based meat alternative market, while cultured meat technology has not yet reached the level for industrialization.

One of the biggest controversies in the development and industrialization of cultured meat and plant-based meat alternatives as meat analogs is the conflict with the traditional livestock industry. Many livestock industries around the world are arguing that the term “meat” should not be used in alternative meat products including plan-based meat alternatives or cultured meat. Plant-based products in the U.S., Europe, and Korea have been prohibited from using meat labels such as “sausage” and “steak” since 2018, on the grounds that consumers might be misled into believing the products were real meat. Several U.S. states and Europe have banned the use of meat labels such as “sausage” and “steak” in plant products since 2018, citing consumers’ misunderstanding that the product is real meat. Additionally, the Korean livestock industry is also demanding that the term “meat” should not be used in meat analogs, such as plant-based meat or cultured meat. This conflict with the livestock industry has a significant impact on meat analog research and industrialization. Therefore, it is predicted that efforts to resolve conflicts with the livestock industries should be prioritized in the industrialization of meat analog in countries around the world.

## FUTURE PERSPECTIVE OF MEAT ANALOG

It can be concluded that there is a high demand for meat analog in the current and future markets. The most significant interest in this product is not due to an increase in vegan consumers; it is driven by consumers concerned about healthy foods and a sustainable environment [1].

Despite receiving much attention around the world and active research, the meat analog market is still in the early stages of growth. In Korea, about 1% of the plant-based meat alternatives sold in the market and cultured meat has not yet been approved for sale. Ismail et al. [1] expected that with current advancements in technology, lab-grown meat with no livestock raising requirement, known as cultured meat, will be expected to boost the food market in the future. Also, insect-based products are promising as the next protein resource for human food. Nevertheless, other than acceptability, cost-effectiveness, reliable production, consistent quality, and product safety are the top priorities. Therefore, regulatory frameworks should be developed [1].

To be commercially successful for large groups of con sumers, alternatives for meat should be highly similar to meat; however, the different nature of plant materials compared to those of meat renders the imitation of meat texture a challenge [85]. Soy protein is known for its cardiovascular disease prevention efficacy, and the FDA has approved the health highlight that states 25 g or more of soy protein a day can reduce the risk of coronary heart disease. In addition, soy protein is the most effective material that can create a taste and quality most similar to traditional meat, therefore it is most widely used as a plant-based meal material. However, soy protein has many disadvantages due to its anti-nutritive factors and potential allergenicity [86]. In fact, the FDA defines milk, eggs, fish, crustaceans, nuts, peanuts, wheat, and soybeans as the main cause of allergies, and wheat and soybeans are the main ingredients for plant-based meat alternatives. Therefore, it will be difficult to claim that plant-based meat alternatives have a greater nutritional advantage than traditional meat. Consequently, this may have a negative impact on the growth of plant-based meat alternative markets in North America or Europe. In addition, gluten is widely used to reproduce the texture of plant-based meat, which is not only inexpensive but also has unique viscoelasticity and adhesion characteristics, making it easy to use to create texture in the products. In particular, wheat gluten is often the basis for imitation meat similar to beef, chicken, duck, fish, and pork. However, gluten is also a food material that consumers avoid as it is known to cause allergies, inflammatory diseases, and autoimmune responses in some people [87]. Therefore, Franca et al [88] also suggested that the health aspects of meat analog ingredients still need improvement. Throughout the development of those products the industry was primarily concerned with taste and texture. Now, however, technological efforts should be directed to nutritional solutions, such as to reduce the amount of saturated fat, maintain micronutrients and other plant protein compounds, and reduce food additives [88]. Consumers should pay attention to product labels and choose products according to their frequency of consumption [88]. Modern structuring techniques for meat alternatives have recently improved their functionality, but it is necessary to focus on the selection of functionality, sensory properties, safety, and appropriate ingredients for the production of meat analogs [89]. In addition, consumer acceptance of meat analogs is highly unsatisfactory, which should be improved through appropriate research and recognition [89].

## CONCLUSION

Interest in meat analog is increasing as interest in health, environment, and sustainability of resources is sharply increasing. In addition, meat analogs are being industrialized in the order of plant-based meat, cultured meat, and ingredients, such as high protein and fat from edible insects. This review found that the research and investment of many companies, including venture companies and academia, are being actively conducted for the industrialization of meat alternatives. Summarizing the current status related to the industrialization of meat analog, studies for plant-based meat, mycoprotein, and edible insects mainly involved sensory properties (texture, taste, flavor, and color resembling meat), nutritional and the safety evaluations, acquisition methods of meat alternatives, and commercialization. Cultured meat is mainly studied to develop muscle satellite cell acquisition and support techniques or materials for the formation of structures. However, it seems that the technologies have not reached the level for active industrialization. Even though there are differences in food categories and labeling between countries, it is common to cause confusion or to relay false information to consumers; therefore, accurate information should be provided. This study suggests that criteria relevant to safety and regulations in meat analog development should be established in the industrialization of meat analog, and that an effort should be made to coexist without conflict with the livestock industry.

## Figures and Tables

**Table 1:** Classification and labeling of alternative foods by country

Classification		South Korea	United States	Europe	China/Japan
Plant protein	Management agency	-Ministry of Agriculture, Food and Rural Affairs, - Ministry of Food and Drug Safety	Food Safety Inspection Bureau (FSIS) of USDA, FDA	European Food Safety Agency (EFSA)	China: State Administration for Market Regulation and National Health Commission Japan: Ministry of Agriculture, Forestry and Fisheries
	Labeling	Use of terms such as “hamburger patties,” “meat,” “nuggets,” and “steaks,” along with plant-based terms such as “veggies,” “vegetarian,” and “soy protein”	Each state has a different notation, but is recommended to use it with terms such as “meat free,” “meatless,” “plant-based,” “veggie-based,” and “made from plants”	The use of names such as “milk,” “cheese,” and “cream” in vegetable dairy products is prohibited. Names indicating the shape and composition of “meat products” such as “steaks,” “sausages,” and “burgers” can be used in plant-based protein foods	“Meat” and “milk” can be used if words similar to “vegetability” are added in a consumer-friendly way
	Classification system	According to the Food Code, “16. Agricultural processed foods” are classified as “16-7. Other agricultural processed foods - (3) Soybean processed products or (5) Other agricultural processed products”	Classified as “subpart D-special dietary” according to the FDA’s Code of Federal Regulations (CFR) It follows the existing classification system, but is classified into a different category from general agricultural products	EU has no separate food classification system	China: Classified as GB2712 <Bean products> according to the existing classification system Japan: Classified as “24. Other processed foods” according to the existing classification system
	Related law	- Fundamental law on Agriculture, Rural Affairs and Food Industry, - Food Code of Ministry of Food and Drug Safety	Code of Federal Regulation, Food Safety Modernization Act, Federal Food, Drug, and Cosmetic Act	General Food Law, European Commission	- China: Corporate Standard T/CIFST based on National Food Safety Standards - Japan: Japanese Agricultural Standards (JAS)
Cultured meat	Management agency	- Ministry of Agriculture, Food and Rural Affairs, - Ministry of Food and Drug Safety	Food Safety Inspection Bureau (FSIS) of USDA, FDA		- China: Ministry of Agriculture and Rural Affairs (MOA) contains the contents of cell culture in the Plan to advance agricultural and rural modernization. - Japan: Ministry of Agriculture, Forestry and Fisheries
	Labeling	It is under discussion by referencing overseas cases depending on whether the cultured meat industry is considered as livestock	Meat terms can be used under the condition that they include “cell cultured products,” “lab-grown,” etc. (some states propose a bill prohibiting the use of meat terms in cultured meat)	No legal definition to date because sales of cultured meat have not been approved	Currently under discussion
	Classification system	If the cultured meat industry is classified as livestock: the possibility of establishing a subcategory of Food Code. If not classified as livestock: the possibility of new types being created.	The USDA and the FDA have reached an official agreement on how to regulate cultured meat	The EU has no separate food classification system	Current related content not confirmed or under discussion
	Related law	Under discussion	Federal Food, Drug, and Cosmetic Act, Federal Meat Inspection Act, Poultry Products Inspection Act	Novel Food Regulation	Current related content not confirmed or under discussions

(Modified from regulations of the Ministry of Agriculture, Food and Rural Affairs; USDA, FDA; European Food Safety Agency; State Administration for Market Regulation and National Health Commission, Ministry of Agriculture, Forestry and Fisheries) This data is the situation at the time the paper was written.

**Table 2:** The global industrialization status of alternative food technologies

Company	Description of products
Impossible Foods (U.S.)	- Developed plant-based hamburger patties using protein from wheat, coconut oil, and meat alternatives, such as potato protein and heme. - To mimic the texture of meat, potato protein is added, and coconut oil is used to mimic meat juice. - It adds a leghemoglobin gene that can be extracted from soybean root lump or genetically engineered yeast cells and combines precision fermentation technology that produces high concentrations of heme by proliferating yeast cells through fermentation. - Soybean leghemoglobin is used as a flavoring agent, and it is used as a colorant due to its red-colored properties. - It applied for a technology patent that uses leghemoglobin, a heme protein derived from plants, to develop alternative meat.
Beyond Meat (U.S.)	- Chicken strips were developed that do not include chicken, but instead use beans, peas, yeast, etc. - It consists of meat alternative proteins, such as peas, mung beans, and brown rice, imitates meat marbling using coconut oil, and develops a “Beyond Meat Burger” patty that imitates meat juice using beet juice. - It applied for a patent for technology related to the development of alternative protein foods similar to meat that have similar structures and textures to meat products (US-2017/0105438-A1). - The U.S. Patent and Trademark Office (USPTO) has registered 108 trademarks of vegetable alternative protein foods, including Beyond Burger, Beyond Beef, Beyond Sausage, etc.
Eat Just (U.S.)	- Its product is based on mung bean protein, and it developed Just Egg, a vegetable egg alternative food with carrots and turmeric added to form the yellow hues of onions and eggs to add flavor. - The mung bean protein in the Just Egg product was selected as the first new soybean protein food additive by the European Food Safety Agency (EFSA) in recognition of its safety in food nutrition and food allergies. - Applied for a technology patent on a vegetable egg substitute and its manufacturing method (US-20140356507-A1).
Fiction Foods (U.S.)	- Developed Performance Scramble products that can replace eggs by applying fermentation technology to Euglena, a nutrient-rich single-celled organism found in fresh water and seawater.
Oatly (Sweden)	- Developed vegetable protein drinks using rapeseed oil and oats that are safe from the GMO controversy. - It applied for a patent related to beta-glucan, a dietary fiber in oats (EP-112441-B1).
Vegan Finest Foods (Netherlands)	- Its product uses tapioca starch, flax, and rapeseed oil. Vegan zeastar, an alternative protein food for vegetable seafood, such as salmon and tuna, was developed.
Aizhiwei (Taiwan)	- Developed dairy products with added glucosamine and *Cordyceps militaris* based on vegetable milk using oatmeal.
QUORN (England)	- A nutritious alternative protein food was developed using mycoprotein produced using the micro mold “*Fusarium venenatum*” with a high protein content. - Applied for a patent related to the manufacture of morphological stability and highly cultivated *Fusarium* strains (US-5980985-A).
Bugfoundation (Germany)	- Developed edible insect burgers that taste similar to sunflower seeds or peanuts using the material of the Buffalo worm (*Alphitobius diaperinus*).
Essento (Switzerland)	- Developed “Mealworm Burger,” an edible insect burger that uses beetle larva to taste like mushrooms, nuts, and shrimp.
Micronutris (France)	- Protein bar products with high protein and essential amino acid content were developed using edible insects such as *Tenebrio molitor* and *Sigillatus*.
Mosa Meat (Netherlands)	- It was founded by Professor Mark Post of the Netherlands, who held the world’s first cell culture tasting event in 2013. - In 2015, after the start of Mosa Meat, the basic concept of cell culture meat production was disclosed. - When muscle satellite cells are separated from the muscle tissue of livestock and cultured to surround pillars made of collagen, the concept of muscle satellite cells differentiating into muscle fibers and taking the form of rings is established. - In 2019, a patent was sought for a method of producing a large amount of muscle fibers in the form of the same ring. - The method is further developed, and the mass production of cultured meat is currently being prepared.
Memphis Meat (Upside Foods) (U.S.)	- It has a method of growing muscle cells in the form of sheets hundreds of micrometers thick after fixing them to auxiliary materials as a key technology. - It has a patented method of thickening the thickness of the cell culture sheet by controlling protein expression, such as yes-associated protein 1 (YAP1) manipulation. - It has a patent for the overexpression of glutamine synthase in cells through genetic manipulation. - Currently establishing a pilot scale culture system.
Meat tech 3D (Israel)	- Its main focus is on producing cell culture meat that combines 3D printing technology. - It is studying a technology that uses food-derived ingredients to cross-differentiate fibroblast or mesenchymal stem cells into fat to obtain animal fat and improve flavor by mixing it with alternative meat.
Future Meat Technologies (Israel)	- Developed a method of cultivating muscles or fats *in vitro* using transdifferentiation. - It has obtained a patent for producing fibroblasts by continuously cultivating fibroblasts derived from chickens, deliberately creating an environment unfavorable to the survival of cells and selecting cells that have survived and changed. - The floating culture method is used for the mass production of cultured meat and the vegetable protein-based support is used for fat differentiation. - Recently, it was announced that it reached the unit price of $66/kg without using fetal bovine serum.
Super Meat (Israel)	- In November 2020, a restaurant was opened to allow the consumer to taste chicken cultured meat. - Currently, a 1,000-liter incubator is in operation. - It does not form muscle fibers and uses cells that are not differentiated as food ingredients. - Super Meat’s tasting product is a hybrid cell culture in which animal cells are mixed with vegetable substitute meat, and the mixing rate of animal cells does not exceed 30%. - Patented using chicken embryonic stem cell lines as starter cells.
Aleph Farms (Israel)	- It has the technology to use textured soy protein, a substance left after squeezing oil from soybeans, as a support for muscle cells. - Various cells derived from livestock are mixed and cultured on the support, suggesting conditions in which the support does not break. - Achieved the first successful differentiation of muscle fibers within a support where three-dimensional growth occurs. - Recently, research on the use of pluripotent stem cells and induced pluripotent stem cells is also underway.
Eat JUST (U.S.)	- Recently, it started a cell culture meat production project and grew into a company with cell culture-related technologies. - With chicken cultivation as the main focus, it became one of the world’s first three companies to obtain a cell-culturing food license in 2020, and it is the only company to carry out sales since then. - It produces cultured meat by floating and culturing immortalized cells and allowing them to be eaten without differentiation into muscle fibers. - It obtained a patent to use both immortalized fibroblasts and naturally immortalized fibroblasts without genetic manipulation through genetic manipulation.
Shiok Meats (Singapore)	- Shrimp Dim sum was approved as food with Eat Just in 2020. - GMO cell lines are used in the experimental stage by producing cell immortality through genetic manipulation. - Mass production is carried out in a floating culture method, and support is not used.
WildType (U.S.)	- The goal is to make salmon cultured meat and release products such as Sushi. - The method of obtaining starter cells from fish eggs and gene manipulation are performed to extend life *in vitro*, and a patent was sought for a product that uses a support produced by electrospinning.

**Table 3:** The industrialization status of alternative food technologies in Korea

Company	Description of products
Lotte Foods	- In April 2019, the company launched the first “Enature Zero Meat” brand while producing “Zero Meat Nuggets” and “Zero Meat Cutlet” based on wheat protein. - It reproduces the muscle fiber of meat with vegetable protein extruded from whole wheat and embodies the chewy texture unique to chicken.
CJ CHEILJEDANG	- Launched “Plantable,” a 100% vegetable product brand certified as vegan. - Using ingredients, such as cabbage, onions, leeks, and vegetable oils, such as canola oil and onion oil, “Bibigo Plantable Wang Gyoja and Kimchi Wang Gyoja” products were developed.
Nongshim	- Launched Veggie Garden, a vegan food brand based on vegetable alternative meat. - It has developed products such as “Veggie Garden Crispy Sweet and Sour Pork,” “Veggie Garden Tender Steak with Sweet Soy Sauce,” and “Veggie Garden Vegetable Cheddar Cheese Slice,” which are made from vegetable proteins such as peas, chickpeas, and coconut oil. - High Moisture Meat Analog (HMMA) has been developed that can implement its own meat-like flavor and texture.
Dongwon F&B	- Dongwon F&B signed an exclusive supply contract with Beyond Meat of the U.S., which manufactures vegetable meat, in December 2018 and introduced the vegetable meat patty “Beyond Burger” to take the lead in the domestic vegan food market. - Since then, Beyond Beef and Beyond Sausage have been additionally released to expand Beyond Meat’s brand lineup. - Beyond Meat is a 100% vegetable alternative meat product made of protein extracted from beans, mushrooms, and pumpkins.
SajoDaerim	- In April 2021, Daelimsun 0.6 Chaedam Dumplings were officially certified as vegan dumplings by the Korea agency of Vegan Certification and Services for the first time in Korea, and the company has been steadily introducing products for vegetarians.
UNLIMEAT	- Developed vegetable slice meat products with low fat content and rich protein by utilizing skimmed bean powder and micrograin, which are by-products of grain processing. - It has acquired its own patented technology related to “protein molding extrusion” that can simulate the texture of meat based on vegetable materials. - Applied for a patent on the composition and production method of grain meat that can provide the same texture as traditional meat (Application No. 10-2008-0090169).
The PlantEat	- Discoverer of its own pure vegetable raw materials, molecular data analysis, and database (DB) for animal food replacement. - It has developed Eat’s Better Mayo product, which is the only vegan certified product in Korea by the Vegetarian Society of the UK, using domestic medicinal beans, rosemary extract, and lemon concentrate as ingredients. - Based on the data, it developed a 100% vegetable alternative milk “XILK” product by mixing natural ingredients, such as soy protein, sugar apple, and coconut oil, which can mimic the taste, color, and nutrition of milk. - A patent was filed for the method of producing clean-labeled soybean powder with excellent quality by suppressing protein denaturation (Application No. 10-2020-0143195).
ALTist	- It launched “Instead of Meat,” a representative vegetable ingredient-based alternative meat brand. - Products such as “vegan marinated ribs,” “vegan jerky,” and “vegan spicy pork stir-fry” are released using vegetable protein extracted from beans and wheat and ingredients, such as dietary fiber, starch, and vegetable oil. - Applied for a patent related to the method of preparing vegetable meat and food composition using soybean without a fishy odor (Application No. 10-2020-0086088).
KEIL	- It has developed alternative protein materials, such as dry powder, protein extraction concentrate, and fat extracted from *Tenebrio molitor* and crickets. - Processing technology was developed to standardize the quality of edible insects using high-temperature hot air and far-infrared rays.
Future Food Lab	- Developed a high-protein powder shake product called “more noble savory shake” using oat powder and high-protein powder. - It launched protein bar products using “Future Protein,” an insect protein manufactured by processing soybean separation protein (ISP), brown *Tenebrio molitor*, crickets.

Lottefoods.co.kr, cjthemarket.com, nongshim.com, xilk.com, eatsbettershop.com, smartstore.naver.com/viomix, keilcorp.com, fflab.kr, dongwonmall.com, sajomall.co.kr, unlimeat.com.

**Table 4:** Current status of alternative food research and industrialization

Alternative food	Sources	Characterization and production
Plant-based meat	Leghemoglobin	- Alternative meat patty made by using leghemoglobin from soybean root nodules. - The developed alternative patty (DAP) showed a similar moisture content as that of the meat patty (MP) and commercial vegetable patty (CVP), which ranged from 53.56% to 53.81%. - The amino acid composition, color, and physical properties in DAP were similar to that of the MP (Kim et al [38]). 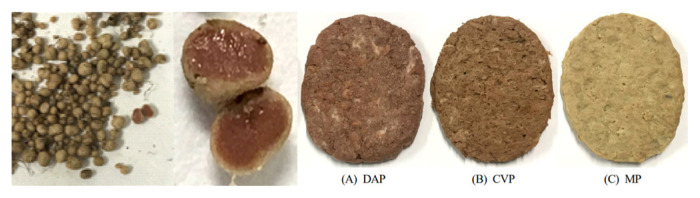
	Leghemoglobin	- Leghemoglobin is extracted from soybean root to produce alternative meat and can be used to mimic the texture of meat (KR patent 10-2021-0097716, 2020).
	Soybean protein mixture	- Alternative meat made by food composition including soybean protein, vegetable protein, gluten, starch, a protein crosslinking agent, and protein hydrolysate. - The shape of the alternative meat is similar to that of the microfiber structure of conventional meat exhibiting excellent functional and nutritional properties (KR patent 10-2020-0070921, 2019). 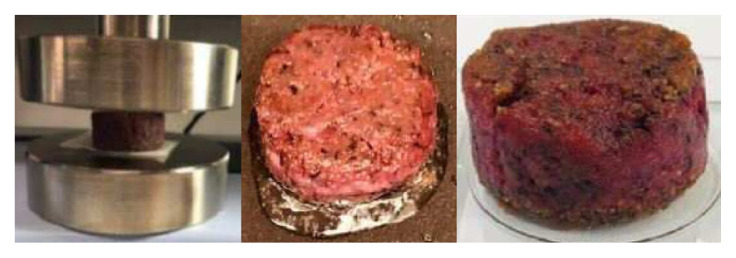
	Bean protein concentrate	- Alternative sausage made by mixed concentrated soy protein, tissue vegetable protein, and tissue soybean protein extracted from soybeans, mung beans, red beans, and peas. - Alternative sausage (A) and partial alternative sausages (B) are similar in flavor and nutritional content as compared to commercial sausage (C) and they have excellent physical properties, textures, colors, and overall appearances (KR patent No 10-2020-0090170, 2020). 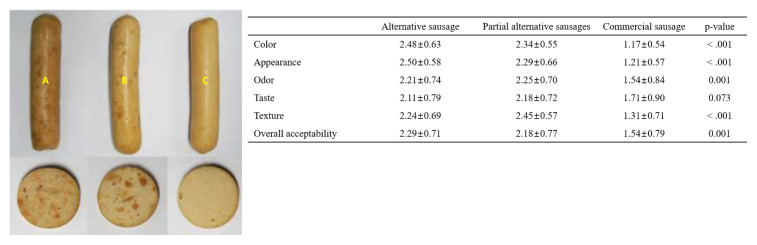
Mycoprotein	Quorn-mycoprotein such as *Fusarium venenatum*	- Production of mycoprotein from agro-industrial wastes extracted by using numerous strains of microorganisms through submerged fermentation and solid-state fermentation. - The difference between submerged fermentation and solid-state fermentation is the moisture level under the medium condition to cultivate strains. - Mycoprotein have high protein levels, fiber, and micronutrients, including cooper, zinc, and selenium (Ahmad et al [42]). 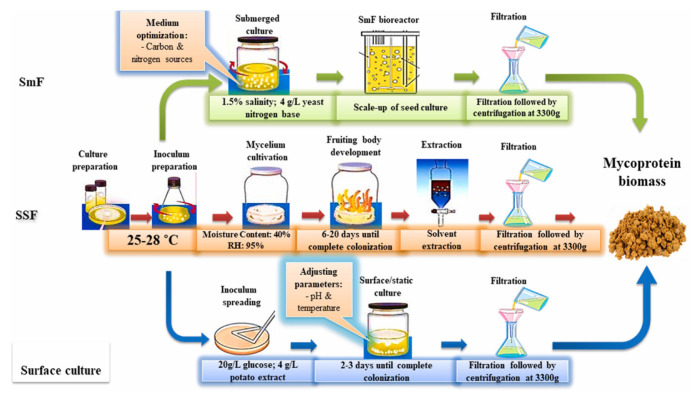
Mycoprotein	Quorn™ with *Fusarium venenatum* A3/5	- Quorn™ fermentation process applied to produce alternative meat product Quorn™. - Mycoprotein obtained by fungus *Fusarium venenatum* A3/5; their process follows the steps (1) fermentation, (2) RNA reduction, (3) centrifugation, and (4) chiller to harvest mycoprotein paste (Meyer et al [20]). 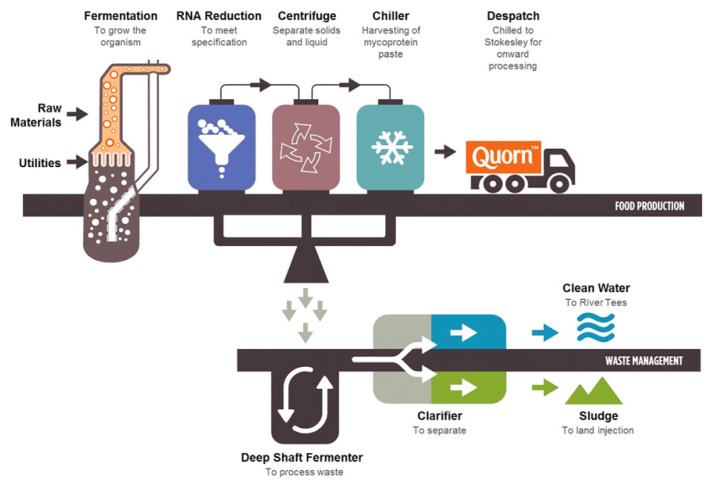
Mycoprotein	Yeast (*Rhodosporidium toruloides*)	- *Rhodosporidium toruloides* contributes to producing carotenoids, which are responsible for the red color of the cells, and supplies antioxidants that are applicable to the food industry (Park et al [56]; Lee et al [57]).
Edible insects	Edible grasshopper (*Schistocerca gregaria*) and honeybee brood (*Apis mellifera*)	- Protein powder obtained from edible grasshopper and honeybee brood was produced by defatting, alkaline, and sonication-assisted extractions. - High amount of protein fractions indicated improved functional properties (Mishyna et al [62]). 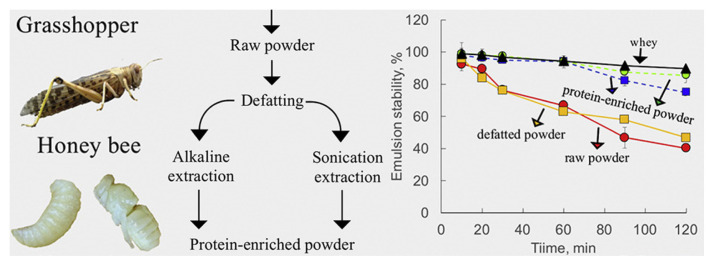
	Edible migratory locust	- Protein hydrolysate obtained from the edible migratory locust was degraded by the mixed enzymes. - The hydrolysate increased in solubility, emulsifying activity, and foamability compared to that of the non-hydrolyzed migratory locust protein (Purschke et al [63]).
	*Protaetia brevitarsis larvae*	- Protein hydrolysate obtained from the edible *Protaetia brevitarsis* larvae was produced using five proteases including alcalase, bromelain, flavourzyme, neutrase, and papain. - Among the proteases, alcalase was higher in low molecular peptides with high antioxidant properties (Lee et al [64]).
	Several species of edible insects	- The edible insects and insect-based food products indicated lower heavy metal, DDT, and dioxin compound concentrations than common animal products (Poma et al [67]).
	Cricket powder	- The cricket powder indicated an increase in the content of the probiotic bacterium (*Bifidobacterium animalis*) and a decrease inflammation factors (Stull et al [68]).
	Yellow mealworm	- 40% pork meat and 10% yellow mealworm made frankfurters an alternative meat product, and the frankfurters were similar to the overall acceptability of the control frankfurters (Choi et al [69]). 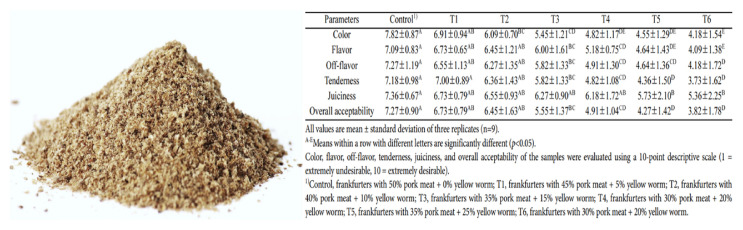
Cultured meat	Muscle satellite cells	- The muscle satellite cells are important components to produce cultured meat, and the biological effects of numerous myokines and cytokines in skeletal muscle were related to proliferation, myogenesis, and myogenic differentiation (Shaikh et al [73]). 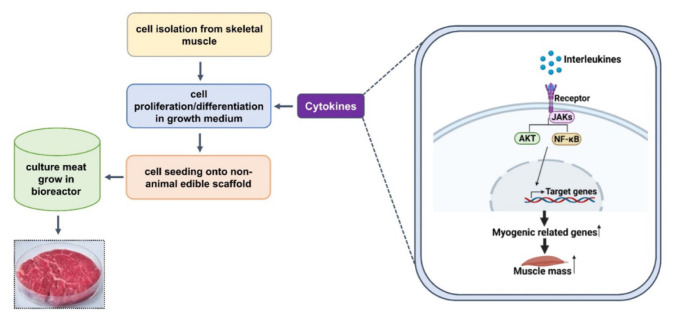
	Bovine satellite cells	- Satellite cells are separated from 2-week-old male calves, incubated in growth medium for 1 week, and then single cell RNA sequencing (scRNA-seq) is performed using the 10× Genomics platform. - Clustering the transcriptomes of 19,096 cultured bovine satellite cells revealed 15 cell clusters with similar gene expression patterns. - The myoblast markers MYOD1, MYF5, and desmin, which are activated and proliferating satellite cells, showed myoblast subsets in clusters 1, 2, 3, and 12 (Lyu et al [74]). 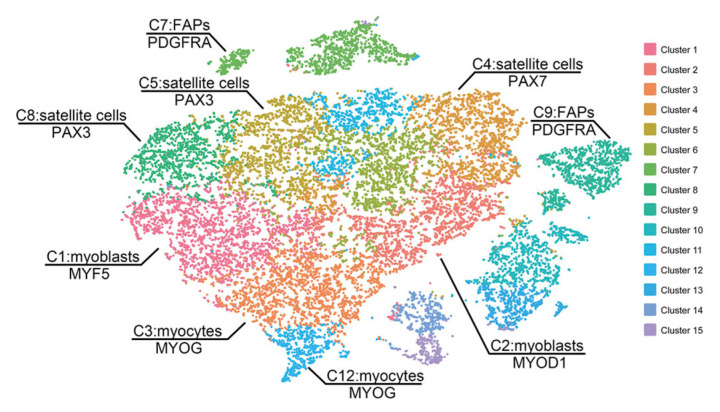
	C2C12 cell	- The metabolism of C2C12 myoblasts and myotubes in serum-free medium (B27, AIM-V) was analyzed and compared with that in conventional serum supplementation culture. - C2C12 myotubes cultured in serum and B27 have prominent glycolytic and oxidative metabolisms, respectively, which are observed in muscle types identified by major histocompatibility complex (MHC) immunostaining. - The metabolic profiles (phosphorylated metabolites and tricarboxylic acid intermediates) in AIM-V culture were similar compared to that of the serum culture (Jang et al [75]). 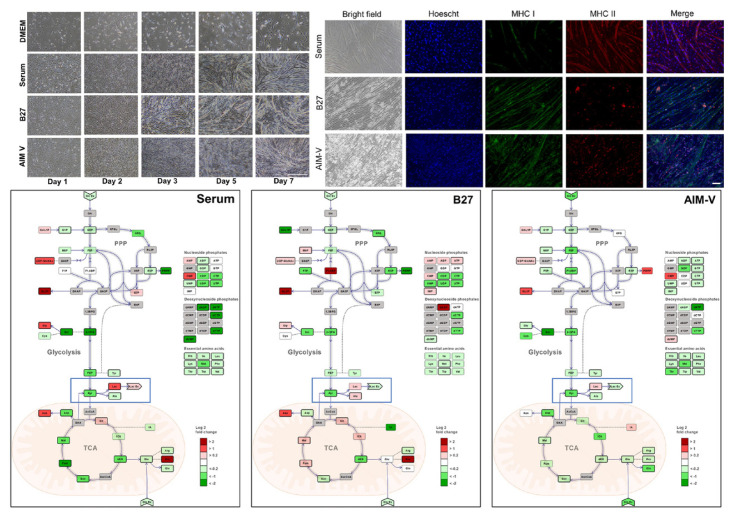
	Serum-free medium with silkworm fibroin	- Serum-free medium was added to silkworm fibroin, which is widely used as an FBS replacer, and the prepared serum-free medium indicated an increase in the cell growth rate without fetal bovine serum (FBS) and can contribute to the income increase of the sericulture industry (KR patent 10-2016-0150130, 2018). 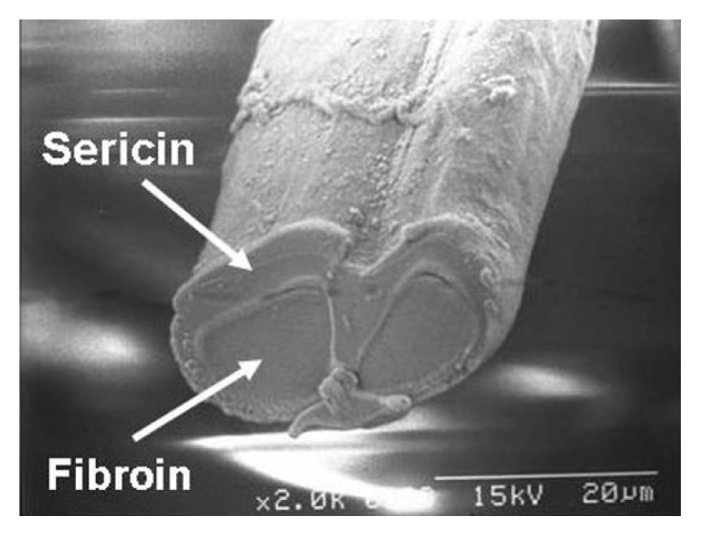
	Four cytokines combination in pig muscle stem cells	- A combination of four cytokines, including LR3-IGF-1, PDGF-BB, bFGF, and EGF, promoted the long-term proliferation of porcine muscle stem cells and reduced the need for fetal bovine serum (FBS) in long-term culture to 5%. - The cytokines were affected by the PI3K/Akt/mTOR and MEK/ERK signaling pathways, which can be utilized to develop cultured meat (Lei et al [78]). 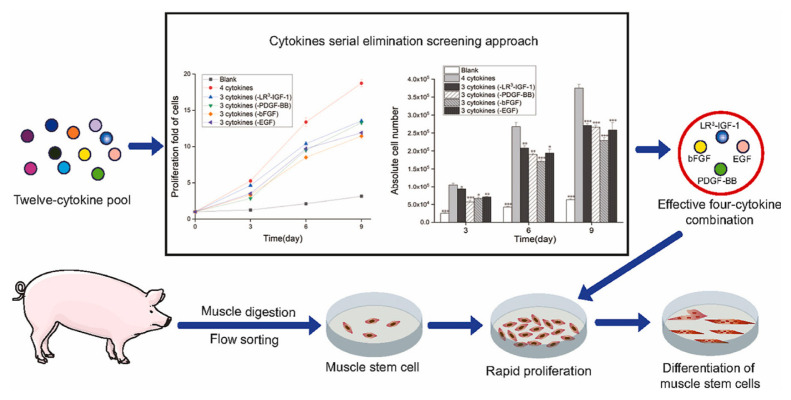
	Satellite cell culture media with mushroom concentrate	- Alternative meat patties were manufactured by spherical granules made with mushroom concentrates and bovine satellite cell culture media using bottom spray under a fluidized coating condition (KR patent 10-2017-0120431, 2018). 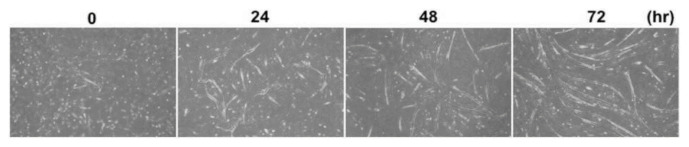
	YAP protein in porcine muscle stem cells	- The yes-associated protein (YAP) protein was found to induce increased cell proliferation and a high degree of differentiation compared to that of pig muscle stem cell control cells and it accumulated in high-density seed muscle stem cells treated with YAP activator lysophosphatidic acid (LPA). - Compositionally activated YAPs with inactive phosphorylation sites promote cell proliferation and stem cell retention in muscle stem cells (Liu et al [79]). 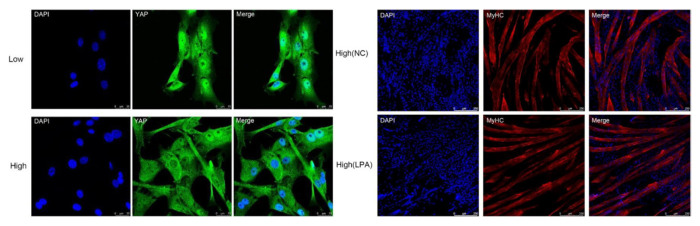
	Decellularized spinach as edible scaffold in bovine satellite cells	- The discovery that decellularization of spinach leaves can produce a network of blood vessels that can potentially maintain the cell’s viability when a bovine’s satellite cells are cultured. - Primary bovine satellite cells are incubated on the surface of the cell-removed spinach leaves and gelatin coated glass for 7 and 14 days. - After 14 days, primary bovine satellite cells seeded from the decellularized spinach leaf scaffold maintain a 99% survival rate. - There is no statistical difference between cells cultured in a gelatin coated dish and cells cultured in decellularized spinach leaf scaffolds (Jones et al [80]).